# Diagnostic Accuracy of Ultrasound and Fine-Needle Aspiration Cytology in Thyroid Malignancy

**DOI:** 10.3390/medicina60050722

**Published:** 2024-04-26

**Authors:** Maria Boudina, Michael Katsamakas, Angeliki Chorti, Panagiotis Panousis, Eleni Tzitzili, Georgios Tzikos, Alexandra Chrisoulidou, Rosalia Valeri, Aris Ioannidis, Theodossis Papavramidis

**Affiliations:** 1 Department of Endocrinology, Theageneio Cancer Hospital, 54636 Thessaloniki, Greece; 2 Department of Surgery, Theageneio Cancer Hospital, 54636 Thessaloniki, Greecepanagiotis.panousis@gmail.com (P.P.);; 3 1st Propaedeutic Department of Surgery, AHEPA University Hospital, Aristotle University, 54636 Thessaloniki, Greece; 4 Department of Pathology, Theageneio Cancer Hospital, 54636 Thessaloniki, Greece

**Keywords:** thyroid malignancy, fine-needle aspiration, ultrasound, thyroid nodule

## Abstract

*Introduction*: Thyroid nodule incidence is increasing due to the widespread application of ultrasonography. Fine-needle aspiration cytology is widely applied for the detection of malignancies. The aim of this study was to evaluate the predictive value of ultrasonography in thyroid cancer. *Methods*: This retrospective study included patients that underwent total thyroidectomy for benign thyroid disease or well-differentiated thyroid carcinoma from January 2017 to December 2022. The study population was divided into groups: the well-differentiated thyroid cancer group and the control group with benign histopathological reports. *Results*: In total, 192 patients were enrolled in our study; 159 patients were included in the well-differentiated thyroid cancer group and 33 patients in the control group. Statistical analysis demonstrated that ultrasonographic findings such as microcalcifications (90.4%), hypoechogenicity (89.3%), irregular margins (92.2%) and taller-than-wide shape (90.5%) were correlated to malignancy (*p* < 0.001). Uni- and multivariate analysis revealed that both US score (OR: 2.177; *p* < 0.001) and Bethesda System (OR: 1.875; *p* = 0.002) could predict malignancies. In terms of diagnostic accuracy, the US score displayed higher sensitivity (64.2% vs. 33.3%) and better negative predictive value (34.5% vs. 24.4%) than the Bethesda score, while both scoring systems displayed comparable specificities (90.9% vs. 100%) and positive predictive values (97.1% vs. 100%). *Discussion*: The malignant potential of thyroid nodules is a crucial subject, leading the decision for surgery. Ultrasonography and fine-needle aspiration cytology are pivotal examinations in the diagnostic process, with ultrasonography demonstrating better negative predictive value.

## 1. Introduction

The incidence of thyroid nodules (TNs) has been increasing over the last years due to the wide use of ultrasonography (US) and other imaging tests. Reportedly, TNs can be found in 2–6% of the population with palpation, in 19–35% on ultrasound and in 8–65% in autopsy series [1]. Since the incidence of thyroid malignancy has been reported to 5.4% for men and 6.5% for women, only a small fraction of those nodules prove to be malignant [2]. Fine Needle Aspiration Cytology (FNAc) is a widely accepted method for the evaluation of TNs and the detection of malignancy with reported accuracy rates varying and exceeding 90% in some reports [3]. FNAc has indisputably contributed to the decrease in the number of unnecessary thyroid surgeries and to the increase of the preoperative diagnosis of malignant thyroid lesions. The 2023 Bethesda System for Reporting Thyroid Cytopathology recommends six reporting categories: (i) nondiagnostic; (ii) benign; (iii) atypia of undetermined significance (AUS); (iv) follicular neoplasm; (v) suspicious for malignancy (SFM); and (vi) malignant [4]. Factors that can influence FNAc results are a possibly inaccessible position of the nodule, operator experience, nodule size and composition, as well as experience in cytology interpretation. 

Many authors have advocated the importance of establishing US criteria for diagnosing thyroid malignancy, which will serve as an additional source of information for the clinician facing a diagnostic dilemma. Certain US characteristics have previously been strongly correlated with malignancy, such as hypoechogenicity, irregular margins, taller-than-wide shape and microcalcifications. In 2015 American Thyroid Association published Management Guidelines for adults with TNs and differentiated thyroid carcinoma, associating sonographic patterns of TNs with the risk of malignancy [5]. In order to assess the risk of malignancy certain sonographic criteria are used. According to ATA, TNs are discriminated as following: TIRADS 1: normal, TIRADS 2: benign conditions, TIRADS 3: probably benign nodules (<5%), TIRADS 4: suspicious nodules (5–80%), TIRADS 5: probably malignant (>80%), TIRADS 6: Biopsy proven malignancy. Risk stratification criteria were conducted by the Korean Thyroid Association (K-TIRADS) [6]. The European Thyroid Association designed another reporting system for ultrasound assessment, EU TIRADS. There are five EU-TIRADS categories: EU-TIRADS 1: normal thyroid lesions, EU-TIRADS 2: benign lesions such us cysts, EU-TIRADS 3: low risk (2–4%) lesions as for isoechoic/hyperechoic nodules with smooth margins, EU-TIRADS 4: intermediate risk (6–17%) lesions such as ovoid, mildly hypoechoic nodules with smooth margins, EU-TIRADS 5: high risk (26–87%) lesions for nodules with suspicious characteristics such as irregular shape or margins, micro-calcifications, taller than wide morphology and markedly hypoechoic solid lesions [7]. Finally, the British Thyroid Association (BTA) classified TNs into 5 categories; U1 = normal thyroid gland, U2 = benign TN, U3 = intermediate/equivocal TN, U4 = suspicious TN, and U5 = malignant TN [8]. 

The aim of this study was to evaluate the predictive value of ultrasonographic features for malignant TNs and to assess the diagnostic performance of these features in thyroid cancer patients.

## 2. Materials and Methods

### 2.1. Study Population

Approval for this retrospective study was obtained from the institutional review board of our hospital (Scientific Council of Theageneio Cancer Hospital, Thessaloniki, Greece, 2661/22 February 2024), and informed consent was obtained from all patients. From January 2017 to December 2022, all patients aged >18 years old who underwent total thyroidectomy in our institution with a histopathology report of a benign thyroid gland or well-differentiated thyroid carcinoma were eligible for the study. Exclusion criteria were insufficient data on the preoperative evaluation of the patients, previous thyroid surgery and a pathology or cytology report of other types of malignancies apart from well-differentiated thyroid cancer.

The studied population was divided into two groups, the malignant group (WDTC-group) which included patients with a histopathological diagnosis of well-differentiated thyroid cancer following thyroidectomy, and the control group (NC-group) consisting of patients with a benign histopathology report. All patients included in this study were preoperatively submitted to a head and neck ultrasound and an ultrasound-guided FNAc (US-FNAc) of the suspicious nodules.

### 2.2. Ultrasonography and US-FNAc

A complete thyroid and neck ultrasound was acquired preoperatively from all patients. The examination was conducted by experienced radiologists (>than 5 years of experience). A US scoring system was implemented in accordance with the 2015 ATA Guidelines, associating thyroid nodules with the risk of malignancy based on their sonographic pattern. The thyroid nodules were assessed regarding the following ultrasonographic features: size, microcalcifications, increased vascularity, hypoechogenicity, taller-than-wide shape, irregular margins, extrathyroidal extension and solid composition. Each of the aforementioned features was appointed one point in the scoring system, reported as US score. Regarding the size, one point was given when the size was more than 10 mm.

A non-aspiration technique using a 23-gauge needle attached to a 5 mL syringe was performed. The samples were evaluated by experienced cytopathologists that were blinded to the US findings. The results of the FNAc were classified into the following six categories according to the Bethesda System for Reporting Thyroid Cytology: (1) nondiagnostic or unsatisfactory (Bethesda System I), (2) benign (Bethesda System II), (3) atypia or follicular lesion of undetermined significance (AUS/FLUS) (Bethesda System III), (4) follicular neoplasm or suspicious for a follicular neoplasm (Bethesda System IV), (5) suspicious for malignancy (Bethesda System V) and (6) malignant (Bethesda System VI). In cases of nodules with both cystic and solid components, an FNAc of the solid part of the nodule was performed. When a multinodular goiter was present, both the largest nodule and the nodule with the most suspicious characteristics were aspirated.

### 2.3. Data and Statistical Analysis

Data were analyzed in the Statistical Package for the Social Sciences 25.0 (SPSS Inc., Chicago, IL, USA) and R Software (version 3.6.2). Relationships with a two-sided *p*-value of less than 0.05 were considered statistically significant. The reference standard for malignancy was the histopathology report. In cases in which both the largest nodule and the nodule with the most suspicious characteristics were aspirated and assessed, the data were analyzed cumulatively, like both nodules being independent. Continuous variables were demonstrated as means with standard deviation (SD) or as medians with interquartile ranges (IQRs), depending on normality having been assumed or not, respectively, while categorical variables were presented as frequencies with percentages (%). The Chi-square test (X^2^) and Fisher’s exact test were applied to investigate the malignancy rate in categorical variables for US features. Independent samples *t*-tests and the non-parametric test of Mann–Whitney were used to evaluate the relationship between continuous variables and malignancies. Univariate and multivariate logistic regression analysis was performed to predict the probability of cancer for both the US score and the Bethesda System. Gender and age at the time of surgery were both included in the final model. After the univariate logistic analysis of every possible factor had been performed, a multivariate logistic analysis was conducted and the final model was built. During univariate regression, a factor was included in the multivariate logistic regression model when it met a statistical significance of a *p*-value less than 0.20. The final model was built using a stepwise backward elimination method with a significance level of 0.05.

In addition, a receiver operating characteristic (ROC) curve was generated to calculate the optimal cut-off point of the US score for thyroid malignancy which was chosen based on the accompanying Youden’s index; sensitivity and specificity were also measured. After the optimal cut-off points for NLR and PLR had been calculated, then the sample size was divided into two groups based on them and the mortality incidence was reassessed [9,10].

## 3. Results

### 3.1. Basic Characteristics

One hundred and ninety-two patients were included in the study. The WDTC group included 159 patients and the NC group included 33 patients. No statistically significant differences were observed in the demographic characteristics between the two groups. Of the total 192 patients included in this study, 162 were female (84.4%) and 30 were male (15.6%). The mean age at the time of the surgery was 52.2 (SD: 13.3) years. The majority of the patients in both groups (*n* = 154, 80.6%) presented with a multinodular goiter (Table 1).

### 3.2. US Features

In total, 246 nodules were evaluated. Nodule size ranged from 8.0 to 58.0 mm, with a median of 21.0 mm (IQR: 17.3 mm). Malignant nodules tended to be slightly smaller in size than benign ones (*p* = 0.005). Microcalcifications were present in 125 (50.8%) of the examined nodules, while the majority of them (113, 90.4%) were proven to be malignant (*p* < 0.001). Hypoechogenicity also showed a high correlation with malignancy (*p* ≤ 0.001), as 89.3% of the hypoechoic nodules represented well-differentiated thyroid carcinomas (134 out of 150 hypoechoic nodules in total), while 61.0% of the malignant nodules were hypoechoic. Hyperechogenicity was rarely observed within both groups. Both irregular margins and taller-than-wide shapes were much more common in malignant nodules (92.2% vs. 7.8%, and 90.5% vs. 9.5%, respectively) and, thus, were highly associated with malignancy (*p* < 0.0001). In addition, almost 62% of the malignant nodules had a taller-than-wide shape (124 out of 201). No correlation was proven between malignancy and the vascularity pattern of the nodules in this study. All of the nodules that demonstrated extra-thyroidal extension on US evaluation proved to be thyroid carcinomas, but it was a rarely noted feature (4.9% of the nodules examined) (Table 2).

### 3.3. Multivariate Regression Analysis and Diagnostic Performances

Multivariate logistic regression analysis was performed to determine the malignancy prediction and the odds ratios for the US score (and its components separately) and the Bethesda System, respectively. In the final multivariate model, gender and age were also included for both models. Independent risk factors for malignancy were independently both US score (OR: 2.177; *p* < 0.0001) and Bethesda System (OR: 1.875; *p* = 0.002) (Table 3 and Table 4). It is interesting to see that the characteristics included in the US score did not contribute equally. The risk score for irregular margins was more elevated than that of “taller-than-wide” or hypoechoic character of the nodule. On the other hand, the nodule size had risk score values near zero.

**Figure 1:**
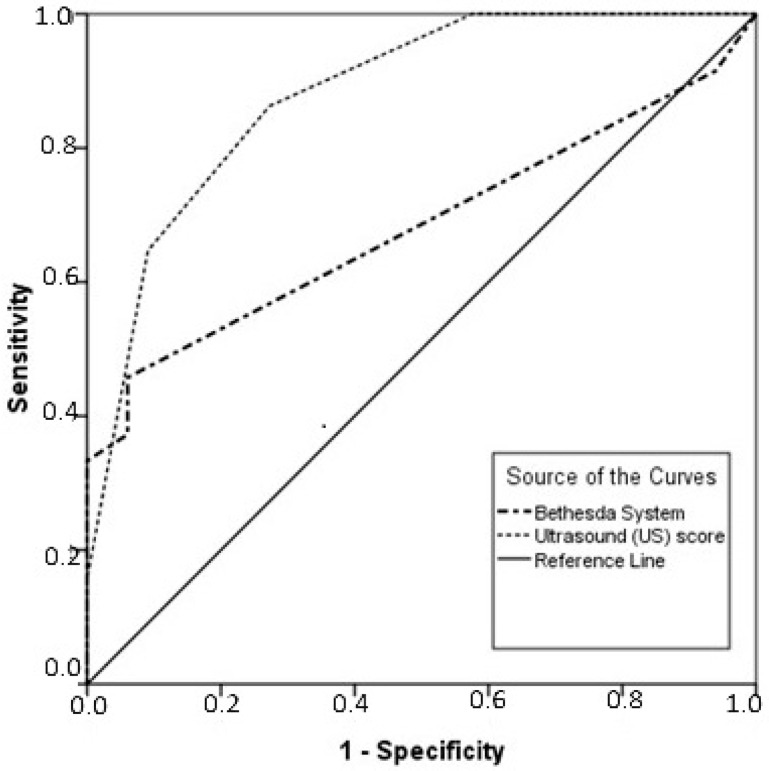
Receiver operating characteristic (ROC) curves of the Bethesda System and US score.

The discriminatory performance of US score for predicting thyroid nodule malignancy and the respective ROC curve is presented in Figure 1. More specifically, the US score exhibited a significant strong discriminatory performance (AUC = 0.784, CI 95%: 0.693–0.875). Regarding the optimal cut-off point of the US score, this was 3.5, with sensitivity of 79.9% and specificity of 66.7%.

### 3.4. Diagnostic Performances

Diagnostic tests were performed to evaluate the sensitivity, specificity, positive predictive value and negative predictive value of both the US scoring system and the Bethesda System in this study.

The two methods were compared in regard to their diagnostic accuracy, sensitivity, specificity and positive and negative predictive value. While the Bethesda System demonstrated specificity and PPV of 100%, its sensitivity was proven to be as low as 33%. On the other hand, the US scoring system was found to have a lower specificity rate and PPV (90.9% and 97.1%, respectively), but with a much higher sensitivity value (64.2%) and a higher NPV (34.5% vs. 24.4%) with a statistical significance of *p* < 0.001 (Table 5). The diagnostic accuracy was 68.75% for the US scoring system and 43.75% for the Bethesda System. A ROC curve analysis was performed for the evaluation of both systems, and the AUC was calculated. The reference standard for malignancy was the histopathology report. When comparing the two AUCs with a Z score test, a statistically significant difference between the two methods was observed. The AUC for the US score ROC curve analysis was 88.0%, a significantly higher value compared to the AUC of the Bethesda System ROC curve analysis with a value of 68.3% (*p* < 0.001) (Figure 1).

## 4. Discussion

The diagnostic evaluation of thyroid nodules is a difficult process. It involves a careful history and clinical examination, followed by a thyroid ultrasound and hormonal tests to assess the thyroid function and the presence of autoantibodies. The clinical importance of thyroid nodules hinges on the need to diagnose thyroid cancer, which occurs in 7–15% of cases based on age, sex, radiation exposure history, family history, smoking habit, obesity and other factors [5,11]. Nowadays, about 40% of the WDTCs diagnosed are less than 1 cm. This tumor shift may be due to the increasing use of ultrasonography or other imaging methods and early diagnosis and treatment [12,13]. In a large retrospective study by Chen et al., the higher incidence of thyroid cancer in thyroid nodules screened with ultrasound rather than palpation was established, and thus, two-thirds of the thyroid nodules believed to be normal were microcarcinomas [13]. The optimization of long-term health outcomes and education about potential prognoses for individuals with thyroid neoplasms is critically important.

In the international literature, there are some systematic reviews and meta-analyses that tried to analyze ultrasound and FNA diagnostic accuracy in thyroid malignancy [14,15]. Ospina et al. concluded that the available evidence only warrants limited confidence on the diagnostic accuracy of FNA due to risk of bias, imprecision and inconsistency among studies, but the likelihood for purely benign and purely malignant potential was found to be high [15]. Regarding ultrasound, Remonti et al. reported that solitary ultrasonographic findings alone could not predict malignancy, but the combination of microcalcifications, a taller-than-wide shape, irregular margins or the absence of elasticity could offer reliable information in terms of malignant potential [14]. The absence of elasticity had the best ultrasonographic performance for malignant results [14]. The study by Ito et al. demonstrated a positive predictive value of ultrasound in 97.2%, while Nie et al. reported ultrasound as being a highly accurate examination for thyroid nodule nature discrimination with a specificity of 33.88% and sensitivity of 92.53% [16,17]. In terms of cytology, FNA was found to have positive predictive value of 100% and negative predictive value of 43.75% in a randomized cross-sectional study [18]. Thus, our study tried to interpret the diagnostic accuracy of ultrasound and FNA in thyroid malignancy diagnosis retrospectively. Our results demonstrated ultrasonographic findings such as microcalcifications, hypoechogenicity, irregular margins and taller-than-wide shapes to be correlated to malignancies with high statistical significance. Furthermore, our study is in concordance with the international available data in terms of positive and negative predictive values of FNA and ultrasound scores, while we concluded that ultrasound specificity was higher and sensitivity was lower than the values proposed in other studies.

Several studies have reported the utility of ultrasonography alone for distinguishing benign from malignant nodules [6,19]. In addition, it is cost-effective, widely available and not invasive. Therefore, ultrasonography has been adopted as the first useful step in determining the location and nature of thyroid nodules [16,20,21,22,23,24]. Alshoabi et al. reported that B-mode ultrasonography alone could differentiate benign nodules with excellent diagnostic accuracy [20]. In this context, we tried to separately examine the sonographic characteristics of thyroid nodules. Their characteristics together with the total US score were analyzed. Moreover, we analyzed the Bethesda score and compared the diagnostic accuracy of the scores. From the uni- and multivariate analysis of both the Bethesda and US scoring systems, we can safely conclude that both could effectively predict the existence of a TN containing a malignancy. Interestingly, the US score displayed higher sensitivity and better negative predictive value than the Bethesda score, while both scoring systems displayed comparable specificities and positive predictive values. Not surprisingly, in the present study, the diagnostic accuracy of the US score was superior to Bethesda score.

When looking closely at the US score and its’ various components, we observed that microcalcification, hypoechogenicity, cystic element, “taller-than-wide” and irregular margins are parameters that are separately strongly correlated with the malignant potential of the TN. This is in consistency with other studies in the literature. Nabahati et al. and Ram et al. proposed the same parameters as indicators of malignancy [19,21]. This means—on a clinical basis—that a patient presenting with one of those characteristics should be considered as having a malignancy and treated adequately.

Moreover, the uni- and multivariate analysis of the logistic regression model of US scoring showed that regardless of the other characteristics, irregular margin, hypoechoic nodule and “taller-than-wide” are strong predictors of malignancy. On the contrary, the nodule size seems not to have a role in the malignant potential of a TN, which is in agreement with Rahimi et al.’s study, concluding that nodule size should not be a criterion for malignancy, but irregular edges, being solid, hypoechogenicity and being a single nodule are major components of malignancy [24]. In the analysis of the Bethesda System, nodule size seems to have a pivotal predictive role in malignancy. Thyroid cytology faces pitfalls in false positive and negative results, such as the misinterpretation of cystic degeneration and squamous cells in partially cystic lesions or the underestimation of architectural and cellular features in follicular patterns, with a wide range of sensitivity (65–99%) and specificity (72–100%) [25]. Zhu et al. resulted that sampling error (86.7%) was the most common cause of false negative diagnoses in FNA, mainly due to nodule size, while interpretation error (80.9%) was the most common cause of false positive diagnoses, affected by overlapping cytological features in adenomatous hyperplasia, thyroiditis and cystic lesions [26]. In our study, the Bethesda System demonstrated specificity and PPV of 100%, while its sensitivity was proven to be as low as 33% and its diagnostic accuracy was 43.75%.

This study has critical clinical implications. First of all, the results of our retrospective study reinforce the fact that ultrasound and FNA biopsy could lead the decision behind the surgical management of suspicious thyroid nodules. Ultrasound has high specificity and positive predictive value for malignant potential, while we found that ultrasound sensitivity is less than already proposed values. FNA biopsy also features high specificity and positive predictive value for thyroid malignancy. Compared to one another, especially in terms of sensitivity and negative predictive values which are lacking in both examinations, ultrasound was found to score higher than FNA biopsy. This is a pivotal new finding that could provide advanced perspectives in the management of suspicious thyroid nodules. In terms of indetermined results between ultrasound and FNA biopsy, we propose that ultrasound findings are more reliable than FNA biopsy of suspicious thyroid nodules, based on the findings of a fully experienced radiologist in the field of the thyroid gland.

## 5. Limitations

A major limitation of our study is the retrospective form of collection of data. We had strict inclusion criteria to lower the risk of selection bias.

## 6. Conclusions

The diagnostic evaluation of thyroid nodules is a difficult process. Sonographic findings dictate fine-needle aspiration cytology and combined approaches lead the diagnostic process of thyroid malignancy. Our study demonstrates that US score has higher sensitivity and better negative predictive value than Bethesda score, while both scoring systems displayed comparable specificities and positive predictive values. Ultrasound seems to be more reliable in predicting thyroid malignancy, with microcalcifications, hypoechogenicity, irregular margins, and taller-than-wide shapes being major factors of malignant potential.

## Figures and Tables

**Table 1:** Basic characteristics of the study population.

	All Patients (*n* = 192)	WDTC Group (*n* = 159)	NC Group (*n* = 33)	*p*-Value
Female Gender (%)	162 (84.4%)	136 (84.0%)	26 (16.0%)	0.331
Mean age at surgery [y] (SD)	52.2 (13.3)	52.1 (13.8)	52.2 (11.0)	0.971
Preoperative median TSH [ng/dL] (IQR)	1.53 (1.60)	1.60 (1.71)	1.71 (1.24)	0.479
Multinodular goiter (%)	154 (80.6%)	125 (81.2%)	29 (18.8%)	0.247

**Table 2:** Ultrasound characteristics of the thyroid nodules.

		All Nodules (*n* = 246)	WDTC Group (*n* = 201)	NC Group (*n* = 45)	*p*-Value
Median nodule size [mm] (IQR)		21.0 (17.3)	20.0 (10.0)	27.0 (60.0)	0.005
Microcalcifications (%)	Yes	125 (50.8)	113 (56.2)	12 (26.7)	<0.001
Hypervascularity of nodule (%)	Yes	155 (63.0)	122 (60.7)	33 (73.3)	0.112
Hyperechoic nodule (%)	Yes	10 (4.1)	3 (2)	7 (15.6)	<0.001
Hypoechoic nodule (%)	Yes	150 (61.0)	134 (66.7)	16 (35.6)	<0.001
Cystic elements (%)	Yes	72 (29.3)	50 (24.9)	22 (48.9)	0.001
Taller than wide (%)	Yes	137 (55.7)	124 (61.7)	13 (28.9)	<0.001
Extrathyroidal Extension (%)	Yes	12 (4.9)	12 (6.0)	0 (0.0)	0.093
Irregular margins (%)	Yes	103 (41.9)	95 (47.3)	8 (17.8)	<0.001

**Table 3:** Logistic models for the US score (WDTC group vs. NC group) prediction.

Parameter	Univariate Analysis					Multivariable Analysis					
	Unadjusted β	SE	OR	95% CI	*p*-Value	Adjusted β	SE	OR	95% CI	*p*-Value	RS
Gender (male/female)	0.257	0.405	1.29	0.59, 2.86	0.525						
Age at surgery (years)	−0.004	0.011	1.00	0.98, 1.02	0.746						
TSH pre-surgery	0.121	0.115	1.13	0.90, 1.41	0.293						
US score	0.788	0.157	2.200	1.616–2.994	<0.001	0.778	0.157	2.177	1.599–2.963	<0.001	
Nodule size	−0.026	0.011	0.97	0.95, 0.99	0.018	0.009	0.017	1.01	0.98, 1.04	0.606	0.01
Taller than wide	2.358	0.353	10.57	5.29, 21.11	<0.001	3.580	0.676	35.86	9.54, 134.83	<0.001	3.6
Irregular margins	2.703	0.372	14.92	7.20, 30.90	<0.001	3.925	0.678	50.67	13.41, 191.54	<0.001	3.9
Hypoechoic nodule	1.504	0.329	4.50	2.36, 8.57	<0.001	2.208	0.561	9.10	3.03, 27.31	<0.001	2.2

Abbreviations: β, coefficient of the explanatory variable; SE, standard error; OR, odds ratio; CI: confidence interval; RS: risk score.

**Table 4:** Logistic models for the Bethesda (WDTC group vs. NC group) prediction.

Parameter	Univariate Analysis					Multivariable Analysis					
	Unadjusted β	SE	OR	95% CI	*p*-Value	Adjusted β	SE	OR	95% CI	*p*-Value	RS
Gender (male/female)	−0.932	0.567	0.39	0.13, 1.20	0.100	−0.976	0.607	0.38	0.12, 1.24	0.108	−0.98
Age at surgery (years)	−0.029	0.013	0.97	0.95, 0.99	0.021	−0.021	0.014	0.98	0.95, 1.01	0.124	−0.02
TSH pre-surgery	0.106	0.130	1.11	0.86, 1.43	0.415						
Bethesda System	0.677	0.207	1.969	1.312–2.951	0.001	0.629	0.484	1.875	1.257–2.798	0.002	
Nodule size	−0.052	0.015	0.95	0.92, 0.98	<0.001	−0.039	0.016	0.96	0.93, 0.99	0.013	−0.04
Taller than wide	−0.029	0.337	0.97	0.50, 1.88	0.931						
Irregular margins	0.782	0.336	2.19	1.13, 4.22	0.020	0.551	0.364	1.74	0.85, 3.54	0.131	0.55
Hypoechoic nodule	0.586	0.367	1.80	0.87, 3.69	0.111	0.406	0.415	1.50	0.67, 3.39	0.328	0.41

Abbreviations: β, coefficient of the explanatory variable; SE, standard error; OR, odds ratio; CI: confidence interval; RS: risk score.

**Table 5:** Diagnostic accuracy of US score and Bethesda System in the diagnosis of cancer.

		Group WDTC	Group NC	Sensitivity	Specificity	PPV	NPV
US score	Positive [3–5]	102	3	64.2%	90.9%	97.1%	34.5%
	Negative [0–2]	57	30				
Bethesda	Positive [5–6]	51	0	33.3%	100%	100%	24.4%
	Negative [1–4]	102	33				
*p*-value				<0.001	0.083	0.083	<0.001

Abbreviations: PPV, positive predictive value; NPV, negative predictive value.

## Data Availability

Data will be available on reasonable request.
